# Supplementary material for: Enabling wider access to human molecular neuroscience research in pain: A simple preservation method for human dorsal root ganglion neurons in Hibernate A media
Source: bioRxiv. 2025 Sep 11:2025.09.06.674648. Preprint. [Version 1] doi: 10.1101/2025.09.06.674648 (PMC12439969; doi:10.1101/2025.09.06.674648)
Supplement: Supplement 1 — Supplemental Table 1 Antibodies and Working Concentrations used for Fluorescently Activated Cell Sorting and Immunocytochemistry Experiments [file media-1.pdf]

University of Texas at Dallas

UTD Donor ID	Age	Sex	Ethnicity	COD	DRG Used	Post Recovery	Technique
UTD-DN0208	36	M	White	Head Trauma/GSW	1x Lumbar	Acute Dissociation	FACS
UTD-DN0240	19	F	White	Anoxia/Asphyxiation/Smoke Inhalation	L4	Acute Dissociation	FACS
UTD-DN0255	44	M	White	CVA/Stroke	2x Lumbar	Acute Dissociation	FACS
UTD-DN0269	29	F	White	Head Trauma/GSW/Suicide	2x T12	Acute Dissociation	Ephys/Ca^{2+} Imaging
UTD-DN0272	19	M	White	Anoxia/Cardiovascular	L2,L3	Acute Dissociation	Ca^{2+} Imaging
UTD-DN0274	20	M	White	Head Trauma/Blunt Injury/MVA	2x L1	Hibernate A	ICC
UTD-DN0278	35	M	White	Anoxia/Drug Intoxication	L1,2x T4	Acute Dissociation	ICC/Ephys/Ca^{2+} Imaging
UTD-DN0284	32	M	White	Head Trauma/MVA	L4	Hibernate A	FACS
UTD-DN0285	23	M	White	Anoxia/Cardiovascular/MVA	L2	Acute Dissociation	ICC/Ephys/Ca^{2+} Imaging
UTD-DN0286	53	M	White	CVA/Stroke	L2	Acute Dissociation	Ephys/Ca^{2+} Imaging
UTD-DN0292	22	M	White	Anoxia/Drug Intoxication	1x Lumbar	Hibernate A	Ca^{2+} Imaging
UTD-DN0292	22	M	White	Anoxia/Drug Intoxication	1x Lumbar	Hibernate A	FACS
UTD-DN0297	44	F	White	Anoxia/Cardiovascular	L4	Acute Dissociation	ICC
UTD-DN0298	46	M	White	Anoxia/Blunt Injury/MVA	2x L2	Hibernate A	Ephys
UTD-DN0301	33	M	White	Head Injury/Blunt Injury/MVA	L2	Hibernate A	Ephys/Ca^{2+} Imaging
UTD-DN0303	18	M	Black	Head Trauma/GSW/Accident	L3	Acute Dissociation	Ephys
UTD-DN0305	31	M	White	Head Trauma/Accident/MVA	2x Lumbar	Hibernate A	Ephys/Ca^{2+} Imaging
UTD-DN0306	28	F	White	Anoxia/Asphyxiation/Suicide	2x S1	Acute Dissociation	ICC
UTD-DN0315	21	M	White	Head Trauma/GSW/Accident	L4	Hibernate A	Ca^{2+} Imaging
UTD-DN0316	28	M	White	Anoxia/Drowning/Accident	2x L5	Acute Dissociation	Ca2+ Imaging
UTD-DN0318	20	M	White	Head Trauma/MVA	2x S1	Acute Dissociation	ICC
UTD-DN0325	46	M	White	Anoxia/Asphyxiation/Accident	2x L2	Hibernate A	Ephys
UTD-DN0327	22	M	White	Head Trauma/GSW/Suicide	2x L3	Acute Dissociation	Ca2+ Imaging
UTD-DN0332	34	F	Pacific Islander	Anoxia/Natural Causes	L2	Acute Dissociation	ICC
UTD-DN0334	20	M	White	Anoxia/Cardiovascular	T5,6,8	Hibernate A	ICC/Ephys/Ca^{2+} Imaging
UTD-DN0341	59	M	White	Head Trauma/GSW/Suicide	T6,T12,L2,L3,L5	Acute Dissociation	Ca2+ Imaging
UTD-DN0346	42	F	White	Anoxia/Drug Intoxication	T10/T11/L5	Acute Dissociation	Ca2+ Imaging
UTD-DN0354	37	M	White	Head Trauma/Blunt Injury/non-MVA	1x Thoracic	Acute Dissociation	Ca2+ Imaging
UTD-DN0354	37	M	White	Head Trauma/Blunt Injury/non-MVA	L1,2,3,5	Acute Dissociation	FACS
UTD-DN0356	19	M	White	Head Trauma/GSW/Homicide	L2	Acute Dissociation	Ephys
UTD-DN0357	34	M	Black	Anoxia/Drug Intoxication	L1,L3	Hibernate A	FACS
UTD-DN0358	31	M	Asian	Anoxia	2x L3	Hibernate A	FACS
UTD-DN0363	29	F	White	Sepsis	L1,L3	Acute Dissociation	FACS
UTD-DN0367	45	M	White	CVA/Stroke	2x Lumbar	Hibernate A	FACS
UTD-DN0371	33	M	White	Head Truama/GSW/Suicide	L2	Acute Dissociation	Ephys
UTD-DN0375	45	M	White	Anoxia/Cardiovascular	2x L4	Acute Dissociation	Ephys
UTD-DN0379	44	F	White	CVA/Stroke	2x L2,L5	Acute Dissociation	Ephys
UTD-DN0388	46	M	White	CVA/Stroke	L2,L3	Hibernate A	FACS

University of Florida

UTD Donor ID	Age	Sex	Ethnicity	COD	DRG Used	Post Recovery	Technique
UTD-DN0322	60	M	White	Head Trauma/Blunt Injury/MVA	2x T12	Dissociated Neurons in Hibernate A	Ephys
UTD-DN0326	39	F	White	Anoxia/Asphyxiation	L2	Dissociated Neurons in Hibernate A	Ephys
UTD-DN0330	41	M	White	CVA/Stroke	1x Thoracic	Dissociated Neurons in Hibernate A	Ephys
UTD-DN0334	20	M	White	Anoxia/Cardiovascular	T7	Dissociated Neurons in Hibernate A	Ephys

Harvard University

UTD Donor ID	Age	Sex	Ethnicity	COD	DRG Used	Post Recovery	Technique
UTD-DN0397	58	F	White	Anoxia/Cardiovasular	2x T11	Dissociated Neurons in Hibernate A	Ephys
UTD-DN0399	24	F	White	Cardiovascular/Overdose	1x Lumbar	Dissociated Neurons in Hibernate A	Ephys
UTD-DN0408	55	M		Anoxia/Cardiovasular	2x T12	Dissociated Neurons in Hibernate A	Ephys
UTD-DN0411	25	F		Anoxia/Cardiovasular	L2,L3	Dissociated Neurons in Hibernate A	Ephys
UTD-DN0418	2	M		Drowning	L3,L4	Dissociated Neurons in Hibernate A	Ephys

Electrophysiology				
Capacitance				
**Group**	**Mean**	**SD**	**n**	**t-test**
Acute	193.95	104.16	118	t_{209}=1.50, p=0.13
Hibernate A	170.12	126.20	93	
Resting Membrane Potential				
**Group**	**Mean**	**SD**	**n**	**t-test**
Acute	-60.11	9.09	114	t_{199}=2.77, p<0.01
Hibernate A	-63.50	7.90	87	
Spontaneous Activity				
**Group**	**% Responders**		**n**	**Fishers Test**
Acute	15.83%		120	p=0.56
Hibernate A	12.50%		96	
Rheobase				
**Group**	**Mean**	**SD**	**n**	**t-test**
Acute	1161.29	941.78	101	t_{181}=1.32, p=0.19
Hibernate A	982.32	881.06	82	
Ramp				
**Group**	**Mean**	**SD**	**n**	**Mann-Whitney**
Acute	3.24	1.53	15	U=76, p=0.14
Hibernate A	2.81	1.06	15	
Amplitude				
**Group**	**Mean**	**SD**	**n**	**t-test**
Acute	96.54	17.68	94	t_{174}=0.28, p=0.78
Hibernate A	95.73	20.69	82	
Half Width				
**Group**	**Mean**	**SD**	**n**	**t-test**
Acute	5.06	2.94	92	t_{172}=2.50, p=0.01
Hibernate A	6.18	2.90	82	
Threshold				
**Group**	**Mean**	**SD**	**n**	**t-test**
Acute	-29.15	8.18	89	t_{167}=0.55, p=0.58
Hibernate A	-28.41	9.23	80	
Rising Slope				
**Group**	**Mean**	**SD**	**n**	**t-test**
Acute	27.41	18.06	94	t_{174}=0.60, p=0.55
Hibernate A	25.91	14.39	82	
Falling Slope				
**Group**	**Mean**	**SD**	**n**	**t-test**
Acute	-5.87	3.85	94	t_{174}=1.09, p=0.28
Hibernate A	-5.34	2.17	82	
After Hyperpolarization				
**Group**	**Mean**	**SD**	**n**	**t-test**
Acute	-47.69	22.82	77	t_{130}=0.96, p=0.34
Hibernate A	-51.54	22.45	55	
**Calcium Imaging**				
20nM Capsaicin % Responders				
**Group**	**% Responders**		**n**	**Fishers Test**
Acute	41.38%		232	p=0.35
Hibernate A	45.45%		101	
20nM Capsaicin Magnitude of Response				
**Group**	**Mean**	**SD**	**n**	**t-test**
Acute	100.43	55.42	96	t_{140}=2.96, p<0.01
Hibernate A	73.47	39.46	46	
20nM Capsaicin AUC				
**Group**	**Mean**	**SD**	**n**	**t-test**
Acute	60.05	40.63	96	t_{140}=1.99, p=0.04
Hibernate A	46.79	27.79	46	

	UTD Acute			UTD Hibernate A			University of Florida Hibernate A			Harvard University Hibernate A		
Measures	Mean	SD	n	Mean	SD	n	Mean	SD	n	Mean	SD	n
Capacitance	193.95	104.16	118	170.12	126.20	93	47.20	19.07	19	146.29	89.31	70
Resting Membrane Potential	-60.11	9.09	114	-63.50	7.90	87	-60.68	4.75	19	-59.57	7.53	49
Rheobase	1161.29	941.78	101	982.32	881.06	82	240.96	165.60	19	896.00	1177.19	70
Amplitude	96.54	17.68	94	95.73	20.69	82	67.78	8.85	19	112.49	17.36	70
Half Width	5.06	2.94	92	6.18	2.90	82	3.30	0.53	19	3.72	2.37	70
Threshold	-29.15	8.18	89	-28.41	9.23	80	-16.30	7.01	19	-22.12	12.64	69
Rising Slope	27.41	18.06	94	25.91	14.39	82	23.75	13.86	19	272.80	199.19	70
Falling Slope	-5.87	3.85	94	-5.34	2.17	82	-3.44	3.78	19	-62.92	48.09	70
After Hyperpolarization	-47.69	22.82	77	-51.54	22.45	55	-41.70	3.92	19	-61.36	6.96	70

Fluorescently Activated Cell Sorting			
**Antibody**	**Company**	**Product #**	**Dilution/Volume**
Myelin Antibodies	Miltenyi	130-096-733	1:10
Zombie UV Fixable Dye	Biolegend	423107	1:100
Human TruStain FcX	Biolegend	422302	5µL
Anti-CD45 (BV605)	Biolegend	304042	5µL
Anti-CD11b (PECy7)	Biolegend	301322	5µL
Anti-CD3 (APC)	Biolegend	300411	5µL
Immunocytochemistry			
**Antibody**	**Company**	**Product #**	**Dilution**
Peripherin	ENCOR	CPCA-Peri	1:1000
Goat Anti-Chicken AF 488	Invitrogen	A11039	1:2000
DAPI	Cayman Chemical	14285	1:5000
